# Structural Color Medical Patch with Surface Dual‐Properties of Wet Bioadhesion and Slipperiness

**DOI:** 10.1002/advs.202203096

**Published:** 2022-09-11

**Authors:** Bin Kong, Rui Liu, Yi Cheng, Yixuan Shang, Dagan Zhang, Hongcheng Gu, Yuanjin Zhao, Wei Xu

**Affiliations:** ^1^ Department of Rheumatology and Immunology Nanjing Drum Tower Hospital School of Biological Science and Medical Engineering Southeast University Nanjing 210096 P. R. China; ^2^ Oujiang Laboratory (Zhejiang Lab for Regenerative Medicine, Vision and Brain Health) Wenzhou Institute University of Chinese Academy of Sciences Wenzhou Zhejiang 325001 P. R. China; ^3^ Department of Orthopedics Tongren Hospital Shanghai Jiao Tong University School of Medicine Shanghai 200336 P. R. China

**Keywords:** bioadhesives, bioinspired, slippery surface, tissue defects, wettability

## Abstract

Developing a self‐reporting bioadhesive patch that has strong adhesion to the wet tissues and meanwhile can avoid adhering to the adjacent tissues is a current research difficulty and challenge. In this paper, inspired by the wet adhesion of spider web, slippery surface of Nepenthes, and structural color phenomena of chameleons, a novel structural color medical patch with surface dual‐properties of wet bioadhesion and slipperiness for internal tissue repair based on inverse opal scaffold is presented. The adhesive surface made by poly(acrylic acid)–polyethylene glycol–*N*‐hydroxysuccinimide ester and gelatin hydrogel can attain tough adhesion to internal wet tissues by absorbing tissue interfacial water and the covalent cross‐linking between the hydrogel and tissue. Besides, the slippery surface made by liquid paraffin infused inverse opal scaffold can avoid adhesion to the adjacent tissues. It is demonstrated that the designed patch can adhere tightly to the defect tissue and improve the tissue repair without adjacent adhesion when applied in a rat model with full‐thickness perforation of the stomach wall. In addition, the responsive structural color can supply a color‐sensing monitoring to evaluate the adhesive and repair process. These features impart the bioinspired patch with great scientific significance and broad clinical application prospects.

## Graphical abstract

Inspired by the wet adhesion of spider web, the slippery surface of Nepenthes, and the structural color phenomena of chameleons, a novel structural color medical patch with surface dual‐properties of wet bioadhesion and slipperiness for internal tissue repair based on inverse opal scaffold is presented.

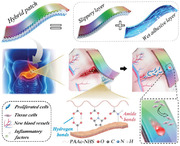

## 1 Introduction

Tissue defects caused by trauma and chronic diseases affect tens of millions of people every year worldwide.^[^ ^1^ ^]^ Surgical sutures are widely used to seal and repair tissues, but they suffer from several inherent drawbacks as they are time‐consuming, require complex operation, and pose a risk of induction of infection and inflammation.^[^ ^2^, ^3^, ^4^, ^5^ ^]^ To avoid the issues and complications induced by surgical sutures, tissue bioadhesives have emerged as promising alternatives to surgical sutures because of their simple and time‐saving operation.^[^ ^6^, ^7^, ^8^, ^9^, ^10^, ^11^, ^12^, ^13^ ^]^ Currently, most tissue bioadhesives exist in the form of glue or liquid, which yield poor adhesion to wet tissues because they are easily diluted by the fluids in vivo. Although solid bioadhesive patches can effectively solve this issue and have demonstrated good tissue adhesion clinically, the property of double‐sided adhesion can result in undesired adhesion to adjacent tissues after surgery. This may further lead to the failure of tissue repair.^[^ ^14^, ^15^ ^]^ In addition, these bioadhesive patches typically lack a monitoring function, which is important for evaluating the efficacy of tissue repair.^[^ ^16^ ^]^ Thus, the development of a novel self‐reporting patch with dual surface properties of wet bioadhesion and anti‐adhesion is highly anticipated.

**Figure 1:**
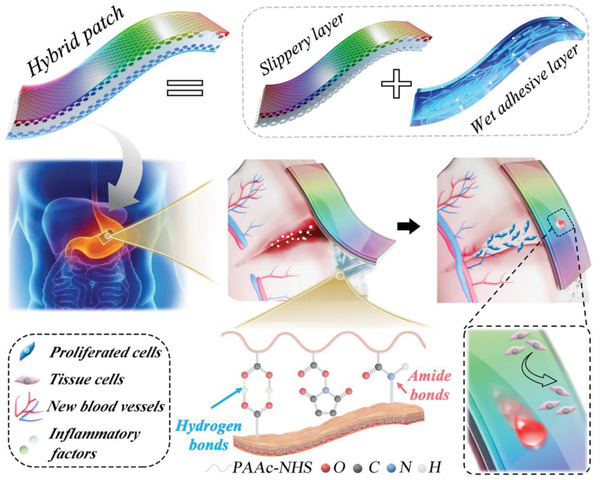
The schematic diagram of the structural color medical patch with surface dual‐properties of wet bioadhesion and slipperiness for gastric tissue repair.

In this study, we present a novel, self‐reporting, structural color medical patch with dual surface properties of wet bioadhesion and slipperiness for internal tissue repair (**Figure** **1**). This patch is inspired by the wet adhesion of spider web,^[^ ^17^ ^]^ the slippery surface of *Nepenthes*,^[^ ^18^ ^]^ and the structural color phenomena of chameleons.^[^ ^19^ ^]^ In nature, the hygroscopic adhesive coat on the surface of cobwebs presents high adhesive toughness, even under highly humid environments. This occurs through moisture absorbance, facilitating the spider to capture its prey. A few reported bioadhesive patches have been inspired by the wet adhesion of spider cobwebs, exhibiting good adhesion under cold or moist environment.^[^ ^20^ ^]^ Further, the rough rim of the *Nepenthes* pitcher plant can lock an intermediary liquid by its microstructure, which then forms a consecutive shrouded liquid film serving as its repellent surface.^[^ ^21^, ^22^ ^]^ Inspired by *Nepenthes*, researchers have developed a slippery liquid‐infused porous surface (SLIPS) for anti‐biofouling, anti‐waxing, and biomedical analyte detection.^[^ ^23^, ^24^, ^25^ ^]^ Furthermore, chameleons can exhibit a remarkable structural color by actively regulating their guanine nanocrystal lattice.^[^ ^19^, ^26^ ^]^ Therefore, various biomaterials with self‐reporting features have been developed based on this mechanism.^[^ ^27^, ^28^, ^29^ ^]^ Although wet adhesives, SLIPS, and structural color have been employed individually in their respective fields, to the best of our knowledge, their combination for a novel structural color patch with anisotropic surface‐based wet adhesion and SLIPS, useful for post‐surgery tissue repair, has not yet been studied.

Herein, we constructed the desired bioinspired structural color hybrid patch with wet adhesion on one side and SLIPS on the other side using an inverse opal poly(lactic‐*co*‐glycolic acid) (PLGA) scaffold. The uniform and periodically arranged nano‐ or micro‐scale porous structure of inverse opal scaffolds can facilitate the infusion of various biomaterials and can endow the patch with an excellent structural color. Medical‐grade liquid paraffin was infused into one side of the PLGA scaffold to form the SLIPS and avoid undesired adhesion with the adjacent tissues after surgery. Acrylic acid (aa), acrylate (polyethylene glycol) *N*‐hydroxysuccinimide (aa‐PEG‐NHS), and gelatin solutions were infused into the other side to form a PAAc–PEG–NHS/gelatin hydrogel (PNH) by radical polymerization. This side served as the layer of wet adhesion wherein PAAc could absorb and remove the liquid at the interface, while NHS could easily cross‐link with the amino group in the tissue to attain tough adhesion. The constructed patch was tested in a rat model with full‐thickness gastric perforation. The patch demonstrated tight adherence to the defective tissue, improved tissue repair without adverse adhesion to the adjacent tissues, and a color‐based sensing to evaluate adhesion and the repair process. Thus, we believe that the proposed adhesive patch will be clinically valuable for internal tissue defects.

## 2 Results and Discussion

**Figure 2:**
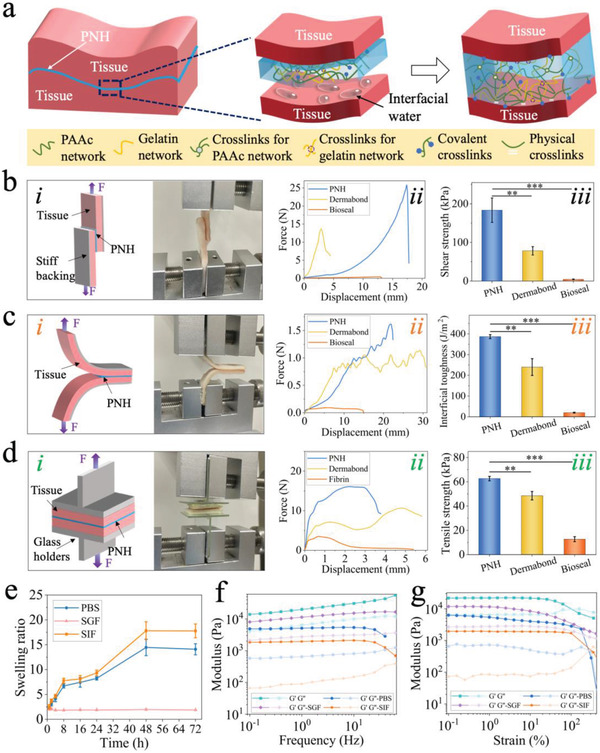
Characterization of the PNH. a) Schematic illustration of the adhesive mechanism of PNH on wet tissues. b–d) Measurement of shear strength, interfacial toughness, and tensile strength of dry PNH, Dermabond, and Bioseal on the porcine skin. i) Schematic illustration and photography showing the adhesion of PNH on porcine skin for tests; ii) the force–displacement curves; iii) histogram of the shear strength, interfacial toughness and tensile strength for (b), (c), and (d), respectively (*n* = 3, biologically independent samples). Data are showed as mean ± SD. **p < 0.01; ***p < 0.001, using the Student's *t*‐test. e) The swelling ratio of PNH after immersing into PBS, SGF, and SIF for specific time (*n* = 3, biologically independent samples). Data are expressed as mean ± SD. f) *G*′ and *G*″ of PNH and PNH when soaking in SIF, SGF, and PBS for 24 h with a frequency amplitude sweep. g) The swelling ratio of PNH after immersing into PBS, SGF, and SIF for specific time (*n* = 3, biologically independent samples). f) *G*′ and *G*″ of PNH and PNH when soaking in SIF, SGF, and PBS for 24 h with a strain amplitude sweep.

To fabricate a tissue‐adhesive hydrogel, acrylic acid (aa), acrylate (polyethylene glycol) *N*‐hydroxysuccinimide (aa–PEG–NHS), and gelatin were mixed, and cross‐linking was initiated under ultraviolet (UV) light. *N*,*N*′‐methylenebisacrylamide (BIS) and 2‐hydroxy‐2‐methylpropiophenone (HMPP) were used as the cross‐linking agent and photoinitiator, respectively. The cross‐linking process and tissue adhesion mechanism of the PAAc–PEG‐NHS/gelatin hydrogel (PNH) are shown in **Figure** **2**a. When exposed to UV light, HMPP creates free radicals, which can induce the opening of double carbon bonds of aa and aa–PEG–NHS, followed by the formation of long PAAc and/or PAAc–PEG‐NHS molecular chains. Meanwhile, the free radicals can also result in the opening of the double carbon bond of BIS, which can covalently cross‐link with PAAc and/or PAAc–PEG‐NHS to form a hydrogel network via a Michael addition reaction. In addition, the molecular chain in gelatin forms a biopolymer network via electrostatic and hydrogen‐bond interactions, as well as covalent cross‐linking between amino groups and NHS. From the ^1^HNMR and mass spectrum results shown in Figure S1, Supporting Information, the molecular weight of aa–PEG–NHS was about 2200 g mol^−1^. The transmittance Fourier transform infrared spectroscopy (FTIR) spectrum of PNH shown in Figure S2, Supporting Information, indicated that the absorption band at 1710 cm^−1^ was attributed to the C=O stretching vibrations of PAAc while 1912 and 1307 cm^−1^ were attributed to the symmetric and asymmetric C—N—C stretching vibrations of NHS, respectively. FTIR results confirmed the successful fabrication of the PAAc‐NHS hydrogel. Then, we determined the molecular weight of the PNH by gel permeation chromatography (GPC), and the average molecular weight Mw was 153k g mol^−1^ (Figure S3a, Supporting Information). Thermogravimetric (TG) analysis was further performed to study the thermal stability of PNH. From Figure S3b, Supporting Information, the mass of the hydrogel changed very slightly when the temperature maintained at 37 °C, indicating that PNH was stable around physiological temperature. Besides, the biodegradation is very essential for the materials applied in tissue engineering and regenerative medicine. Thus, we determined the biodegradation of PNH in the disodium hydrogen phosphate and citric acid buffer with the pH of 7, 5, and 3 for 10 days at 37 °C, and the pH 7 buffer added with collagenase was measured as well. From Figure S4, Supporting Information, the pH of the buffer had little influence on the biodegradation of the hydrogel. With the addition of the collagenase, the hydrogel could be totally degraded within 10 days. When PNH was in contact with the wet tissues under gentle pressure, the presence of carboxylic acid groups in the PAAc allowed quick hydration and swelling of PNH to remove the interfacial water.^[^ ^17^ ^]^ Consequently, PNH could tightly adhere to various wet tissues under the 1) electrostatic interaction and hydrogen bonding between the carboxylic acid groups and the surface of tissues, and 2) the covalent cross‐linking between the NHS and the amino groups on the tissues.

PNH was completely dried to form dry PNH, and the tissue adhesion ability of PNH and dry PNH on porcine skin was evaluated using the lap shear test (Figure 2b, according to the standard method ASTM F2255), 180‐degree peel test (Figure 2c, ASTM F2256), and tensile tests (Figure 2d, ASTM F2258). Commercially available cyanoacrylate‐based (Dermabond) and fibrin‐based (Bioseal) adhesives were used as controls. Dry PNH exhibited a shear strength of 183.5 ± 31.6 kPa, which was higher than PNH (53.4 ± 14 kPa), Dermabond (78.3 ± 10.9 kPa), and Bioseal (4.4 ± 0.7 kPa; Figure 2b and Figure S5a, Supporting Information). In the 180‐degree peel test, the dry PNH showed an adhesion energy of 836.6 ± 9.8 J m^−2^, which was higher than that of the PNH (290.2 ± 13.3 J m^−2^), Dermabond (240 ± 40.6 J m^−2^), and Bioseal (20.1 ± 2.1 J m^−2^; Figure 2c and Figure S5b, Supporting Information). In the tensile test, the dry PNH showed a tensile strength of 62.6 ± 1.9 kPa, which was higher than that of PNH (32.2 ± 2.3 kPa), Dermabond (48.5 ± 3.5 kPa), and Bioseal (12.7 ± 2.1 kPa; Figure 2d and Figure S5c, Supporting Information). The better wet tissue adhesive ability of dry PNH resulted from a faster removal of interfacial water as compared to that of PNH. Therefore, the following experiments were all based on dry PNH.

The swelling ratio of PNH was measured in various biological fluids (simulated intestinal fluid [SIF], simulated gastric fluid [SGF], and phosphate buffer saline [PBS]) as shown in Figure 2e, to evaluate its ability of in vivo wet tissue adhesion. Within 1 h, the swelling ratio of PNH reached 2.5 ± 0.5, 2.3 ± 0.2, and 2.0 ± 0.1 in SIF, PBS, and SGF, respectively. This increased further to 17.8 ± 1.4 in SIF and 14.1 ± 1.1 in PBS, while slightly changing to 1.9 ± 0.02 in SGF, after 72 h of immersion. The difference in the swelling behavior may be attributed to the variation in the pH of the fluids, as a high pH would result in a greater swelling ratio, according to a previous study.^[^ ^6^ ^]^ The swelling ratio of PNH in SIF was greater than that in PBS, which may be attributed to the higher ionic strength of the SIF solution. After immersion in SIF, PBS, and SGF for 24 h, the microscopic structure and mechanical properties of PNH were determined by scanning electron microscopy (SEM) and rheology. As shown in Figure S6, Supporting Information, PNH had a relatively uniform porous structure with an average diameter of ≈5 µm. Immersion in biological fluids led to an increase in pore size, with average diameters of 6, 9, and 15 µm in SGF, PBS, and SIF, respectively. The largest pore size of PNH in SIF was attributed to its highest swelling ratio in this fluid. The values of modulus of PNH before and after immersion in SIF, PBS, and SGF were plotted as functions of frequency and strain (Figure 2f,g). The modulus–frequency curves showed that the storage modulus (*G*′) of PNH under all conditions was larger than the loss modulus (*G*″). Further, the modulus of all PNHs showed minimal changes throughout the frequency range (0.1–10 Hz), indicating that PNH could maintain the hydrogel structure and had long‐term network stability even after immersing in PBS, SGF, or SIF. In addition, the *G*′ of PNH was the largest, followed by that of PNH in SGF and PBS, while *G*′ of PNH in SIF was the smallest. This suggests that immersion in fluids can lower the cross‐linking density of the hydrogels. The modulus–strain curves revealed that when the strain was lower than 100%, the modulus of all the PNHs remained constant, and *G*′ was larger than *G*″ indicating that all the hydrogels had a stable network structure within this strain range. *G*′ of all PNHs decreased with increasing strain, ultimately reaching a value lower than *G*″, suggesting that the network structure of the hydrogels had been destroyed. Finally, the yielding modulus of PNH was the largest, followed by PNH in SGF and PBS, and SIF. This indicated the variation in mechanical strength before and after immersion in the fluids, and was consistent with the swelling and SEM results.

**Figure 3:**
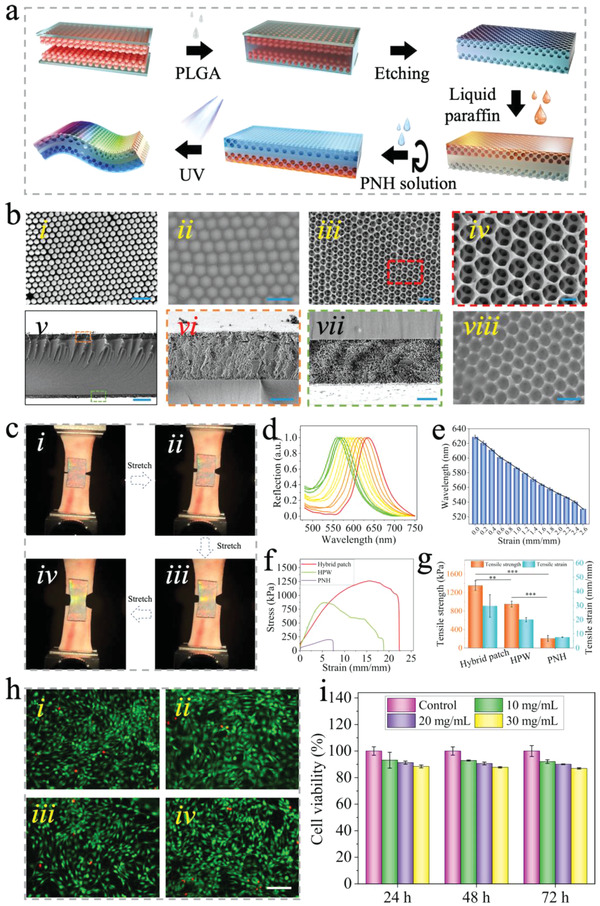
Fabrication and characterization of the hybrid patch. a) Schematic illustration showing the fabrication process of the hybrid patch. b) The SEM images of i) colloidal crystal template, ii) PLGA solution infused colloidal crystal template, iii) PLGA inverse opal scaffold, iv) the area marked with red frame in (iii) at higher magnification, v) the double‐layered PLGA inverse opal scaffold, and the enlarged images are shown in (vi) and (vii); viii) liquid paraffin infused PLGA inverse opal scaffold. The scale bar in (i)–(iii) and (viii) is 500 nm, (iv) is 200 nm, (v) is 20 µm, (vi) is 5 µm, and (vii) is 3 µm. c) The structural color variation during the stretching process of the hybrid patch. d) Corresponding reflection spectra change of the hybrid patch in (c). e) The peak position of the reflection spectra as the function of strain (*n* = 3, biologically independent samples). Data are expressed as mean ± SD. f) The tensile strain–stress curves of the hybrid patch, hybrid patch without inverse opal structure (HPW), and PNH when adhered on porcine skin. g) The corresponding tensile strength and strain of the scaffolds in (f) (*n* = 3, biologically independent samples). Data are expressed as mean ± SD. **p < 0.01; ***p < 0.001, using one‐way ANOVA followed by post‐hoc test. h) The Live/Dead staining images of 3T3 cells after culturing in the extracted liquid of the hybrid patch with the concentrations of i) 0, ii) 10, iii) 20, and iv) 30 mg mL^−1^ for 24 h. The scale bar is 100 µm. i) The cell viability in the extracted liquid after culturing for 24, 48, and 72 h (*n* = 3, biologically independent samples). Data are expressed as mean ± SD.

Although PNH exhibited high adherence to wet tissue, the double‐sided adhesive may result in undesired adhesion to the adjacent tissues after the surgery, further leading to the failure of tissue repair. Thus, we proposed the fabrication of a novel hybrid patch film with dual surface properties of wet bioadhesion and slipperiness by filling the PNH pregel solution and liquid paraffin into a double‐layered PLGA inverse opal scaffold, as shown in **Figure** **3**a. In a typical experiment, silica nanoparticles with the diameter of 295 nm were deposited on the surface of two glass slides to form colloidal crystal templates with ordered structures via self‐assembly of the nanoparticles after the evaporation of the solvent^[^ ^30^ ^]^ (Figure 3b[i]). The interconnected structure of the nanopores in the colloidal crystal template facilitated the infiltration of solutions. Owing to its good biocompatibility and controllable degradability,^[^ ^31^, ^32^, ^33^ ^]^ PLGA was selected as an ideal scaffold material. PLGA solution with a concentration of 20 wt% was filled into the colloidal crystal templates with a distance of 100 µm between the two glass slides. Under capillary action, the PLGA solution easily filled the voids between the orderly distribution of nanoparticles (Figure 3b[ii]), and solidified after the evaporation of chloroform. Free‐standing PLGA scaffolds with a double‐layered inverse opal structure were achieved by immersing them in a 4% hydrogen fluoride solution for 2 h to etch the silica nanoparticles (Figure 3b[iii–vii]). Liquid paraffin, a widely utilized lubricant in the clinic,^[^ ^34^, ^35^ ^]^ was infiltrated into one side of the PLGA scaffolds to impart excellent anti‐adhesive properties, as inspired by the structure of the *Nepenthes* pitcher plant to form a SLIPS (Figure 3b[viii]). Remarkably, the ordered distribution of the silica nanoparticles imparted a colloidal crystal template with unique photonic bandgap properties, leading to a template with a vivid structural color and characteristic peak, as shown in Figure S7, Supporting Information. The characteristic peak of the template assembled by 295 nm silica nanoparticles was at ≈640 nm, blue‐shifted to ≈525 nm for the PLGA inverse opal scaffold (PLGA IO), and further red‐shifted to ≈620 nm after filling with paraffin. Eventually, the PNH pregel solution was filled into the other surface of the PLGA inverse opal scaffold to impart wet tissue adhesion, after polymerization under UV light, to form a film of hybrid patch with structural color and dual properties.

The mechanical strength of the PLGA scaffold, PLGA IO, and hybrid patch were measured after the fabrication process, as shown in Figure S8, Supporting Information. The introduction of the inverse opal nanoporous structure would lower the maximum tensile stress, strain, and Young's modulus of the PLGA scaffold. The introduction of PNH further decreased the maximum tensile strength but improved the elongation at break of the scaffold, indicating that the hybrid patch had good ductility and sufficient flexibility, making it suitable for sensing applications. The structural color of the hybrid patch, based on the inverse opal structure, imparted photonic sensing capability via the dynamic variation of colors. To verify this, we performed a mechanical tensile test by adhering the hybrid patch to porcine skin. First, a hybrid patch without liquid paraffin infiltration was used to carry out the test, as shown in Figure S9, Supporting Information. This resulted in a gradual color change from green to cyan, and the blue‐shift of the reflection spectrum from 536 to 475 nm. After infiltration with paraffin, the hybrid patch exhibited a gradual color change from orange–red to yellow and then to green after stretching, as shown in Figure 3c and Movie S1, Supporting Information. The reflection spectrum was blue‐shifted from 628 to 530 nm (Figure 3d,e). The dynamic variation of colors resulted from the gradual decrease in the interplanar distance of the diffracting planes during the stretching of the patch. To further determine the stretching strength of the hybrid patch when adhering to porcine skin, a tensile test was carried out, wherein a PNH and hybrid patch without an inverse opal structure were used as controls. As shown in Figure 3f,g, PNH exhibited a tensile strength of 206 ± 64 kPa and tensile strain of 7.6 ± 0.2 on porcine skin, and the introduction of PLGA largely increased the tensile strength and strain of PNH. Besides, the tensile strength of the hybrid patch significantly increased when compared with the HPW (*p* < 0.01), suggesting that the presence of interconnected voids in the inverse opal structure enhance the integrity of the hybrid scaffold. To verify the biocompatibility of the hybrid patch, the liquid extracted from the patch, at concentrations of 10, 20, and 30 mg mL^−1^, was used to culture NIH‐3T3 cells. Notably, the remaining unreacted monomers of aa within the PNH were toxic to the cells. Thus, to reduce the content of unreacted aa monomers to the maximum extent, aa was purified before use, and oxygen in the pregel solution was removed before the cross‐linking process under UV light. The 3T3 cells were cultured in plastic culture dishes in normal culture media (control) and in the liquid extracted from the patch at three concentrations for 24, 48, and 72 h, respectively. The Live/Dead staining and Cell Counting Kit‐8 (CCK‐8) assay results, as shown in Figure 3h,i, and Figure S10, Supporting Information, revealed that cell viability slightly decreased with increasing extract concentration. However, cell viability remained over 80% under all conditions, indicating that the hybrid patch had good biocompatibility.

**Figure 4:**
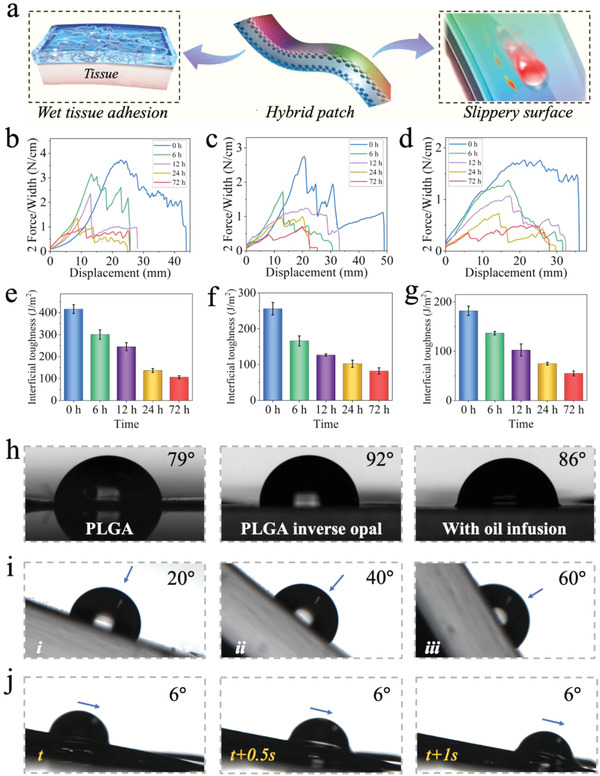
The dual‐properties of wet bioadhesion and slipperiness of the hybrid patch. a) The schematic diagram showing the dual‐properties of the hybrid patch. b–d) The peeling curves of the hybrid patch after adhering on the porcine, stomach and intestine for 0, 6, 12, 24, and 72 h, respectively. e–g) The interfacial toughness of the hybrid patch after adhering on the porcine, stomach, and intestine for 0, 6, 12, and 24 h, respectively (*n* = 3, biologically independent samples). Data are showed as mean ± SD. h) The contact angles of the water droplet on the PLGA scaffold, PLGA inverse opal scaffold, and hybrid patch infused with paraffin oil. i) Images showing the water droplet keeping still on the PLGA inverse opal scaffold with the surface rotating from 0° to 60°. j) Images showing the sliding process of the water droplet on the slippery surface of the hybrid patch with the sliding angels of 6°.

To determine the dual properties (wet bioadhesion and slipperiness) of the hybrid patch, the adhesive strength of the adhesive surface was measured on wet porcine skin, stomach, and intestine followed by determination of the water contact and sliding angles in the different scaffolds (**Figure** **4**a). A 180‐degree peel test was used to determine the adhesive interfacial toughness, as shown in Figure 4b–g. The instant adhesive strengths of the hybrid patch on wet porcine skin, stomach, and intestine were 415 ± 20, 203 ± 9, and 182 ± 10 J m^−2^, respectively. Next, the adhesive strength was measured at 6, 12, and 24 h after adhesion. Although the interfacial toughness on all tissues gradually decreased with increasing adherence time, it was maintained at 106 ± 6, 82 ± 9, and 55 ± 5 J m^−2^ on porcine skin, stomach, and intestine after adhering for 72 h. Therefore, these results indicated that the hybrid patch presented a strong and lasting adhesive ability for various wet tissues. As shown in Figure 4h, the water contact angle of the PLGA scaffold was ≈79°, which increased to ≈92° after the formation of the inverse opal structure. However, perfusion of liquid paraffin decreased the contact angle to ≈86°. In addition, the water droplet in the PLGA inverse opal kept still even after rotating the scaffold at 60° (Figure 4i). However, the filling of liquid paraffin facilitated the sliding of the water drop with a sliding angle of 6°, indicating that the hybrid patch had superior slippery properties (Figure 4j). These results verified the excellent flexibility of the hybrid patch and its effective dual‐properties of wet bioadhesion and slipperiness.

**Figure 5:**
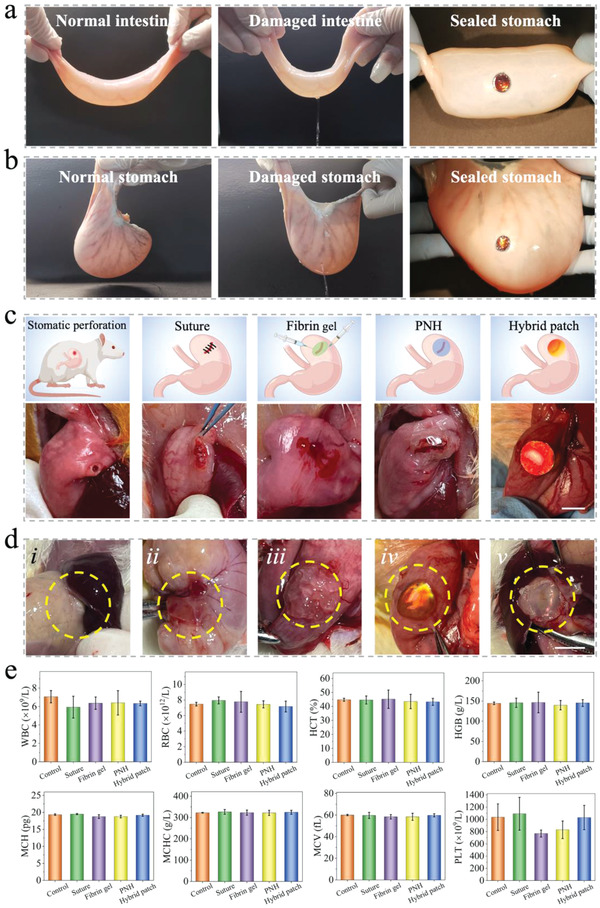
The wound sealing and evaluation of the hybrid patch in vitro *and* in vivo. The hybrid patch with brilliant structural color could effectively seal the perforation with a diameter of 10 mm on the a) porcine intestine and b) rabbit stomach. c) The schematic illustration and photographs of the stomatic perforation, and sealed with suture, fibrin gel, PNH, and hybrid patch. d) The photographs showing the wound area 7 days (i: suture, ii: fibrin gel, iii: PNH, v: hybrid patch) and 3 days (iv: hybrid patch) after the operation. e) The blood routine examination of the blood from the normal rats (control), rats sealed with suture, fibrin gel, PNH, and hybrid patch (*n* = 3, biologically independent samples). Data are showed as mean ± SD.

To further verify the wet adhesion ability of the hybrid patch, porcine intestine and rabbit stomach were freshly harvested, as shown in **Figure** **5**a,b. Distilled water was perfused into the normal intestine and stomach, and no fluid leakage was observed. A perforation was made in the intestine and stomach, and fluid leakage was observed. Then, the hybrid patches were applied on the area of the perforated tissue, as shown in Figure 5. The hybrid patch exhibited a bright structural color, and the fluid leakage was fully inhibited, suggesting that the hybrid patch had good wet adhesion ability in a practical trial. To simulate the static status of the stomach and intestine after surgery, the hybrid patch was sealed on freshly harvested rat tissues, followed by immersion of the sealed stomach tissue into SGF, and the sealed intestinal tissue into SIF for 4 h. The hematoxylin–eosin staining (HE) results shown in Figure S11, Supporting Information, revealed that the adhesion of the hybrid patch caused little damage to the tissues while maintaining the serosal layer of the stomach and intestine intact and the structures similar to the normal tissues without adhesion.

After the in vitro characterization and evaluation of the hybrid patch, we further verified its performance in vivo in a rat model of gastric perforation by excising a perforation with a diameter of 3 mm at the fundus of the stomach. To assess the tissue repair efficacy of the hybrid patch, 1) suture, 2) the commercially available Bioseal, with fibrin gel as the main component, one of the most widely utilized prosthetic biomaterials for the repair of gastric perforation, and 3) PNH, were chosen as controls (Figure 5c). The suture, fibrin gel, PNH, and hybrid patch were used to seal the perforation in four groups of rats, resulting in no observable fluid leakage in any of the groups. 7 days after surgery, the restored states of the sealed area were observed, and an additional observation was carried out for the hybrid patch to study the variation in structural color at day 3. As seen in Figure 5d, the apparent adhesion of the sealed area with the adjacent liver and/or spleen was observed in the suture, fibrin gel, and PNH groups. The HE staining results shown in Figure S12, Supporting Information, clearly demonstrate the post‐surgery adhesion. However, with the hybrid patch, the SLIP surface successfully prevented undesired adhesion with other tissues. In addition, the structural color of the hybrid patch gradually changed from red–orange to yellow. Further, it was barely observed on day 7 owing to the variation and destruction of the porous structure, indicating that the PLGA membrane of the hybrid patch can be gradually degraded.

**Figure 6:**
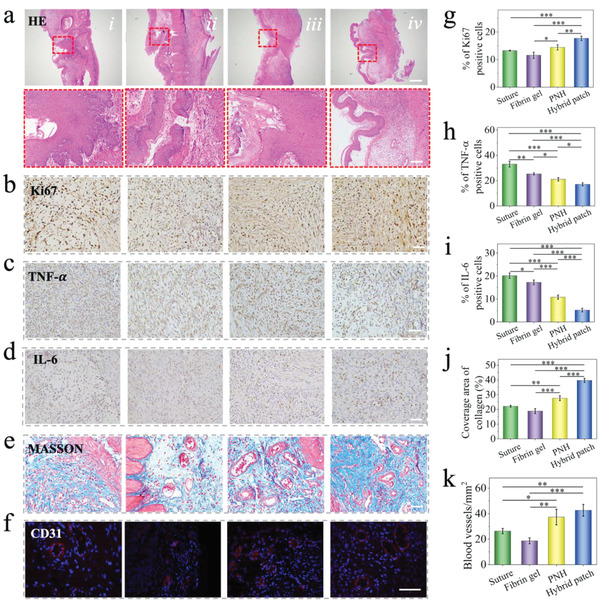
The in vivo evaluation of the hybrid patch on a rat gastric perforation model. Representative a) HE staining, b–d) Ki67, TNF‐*α*, and IL‐6 immunohistochemistry staining, e) Masson staining, and f) CD 31 immunofluorescence staining images of the wound area after treating with i) suture, ii) fibrin gel, iii) PNH, and iv) hybrid patch for 7 days, respectively. The scale bar in (a) is 500 µm, in the enlarged images is 100 µm. The scale bar in (b)‐(f) is 50 µm. g–i) Quantification of Ki67, TNF‐*α*, and IL‐6 positive cells. j) Quantification of collagen coverage area. k) Quantification of CD31 labeled blood vessels. *n* = 3, biologically independent samples. Data are showed as mean ± SD. *p < 0.05; **p < 0.01; ***p < 0.001, using one‐way ANOVA followed by post‐hoc test.

7 days after the operation, the main tissues of the rats in the groups sealed with PNH and hybrid patch, including the heart, liver, spleen, lung, and kidney, were harvested and stained with HE to evaluate the in vivo biocompatibility of our patch. In addition, the PNH and hybrid patch were embedded into the subcutaneous area of the rats for 14 days before HE staining of the main tissues to assess the long‐term in vivo biocompatibility, as shown in Figure S13, Supporting Information. The main tissues were not influenced by patch implantation when compared to the tissues of normal rats. Moreover, routine examination and biochemical analysis of the blood from the four groups of rats and normal rats (control) were conducted to further determine biocompatibility. As shown in Figure 5e and Figure S14, Supporting Information, the index of blood exhibited no significant differences among the five groups. After determination of biocompatibility, the sealed areas of the stomach in the four groups were obtained and subjected to HE, Masson, immunofluorescence, and immunohistochemical staining to further evaluate the repair of the perforated tissue. The HE and Masson staining results of the stomach tissues sealed with suture (i), fibrin gel (ii), PNH (iii), and hybrid patch (iv) are shown in **Figure** **6**a–e. Perforated areas were easily identified, which had been partly repaired in the suture, fibrin gel, and PNH groups. In contrast, the repair in the hybrid patch group was mostly complete with distinct multilayer structures and significantly improved collagen secretion (*p* < 0.001, Figure 6j).

Cell proliferation and local inflammatory response in the wound area were visualized via immunohistochemical staining for Ki67, TNF‐*α*, and IL‐6 (Figure 6b–d and Figure S15, Supporting Information). The Ki67‐positive cells of the hybrid patch group were significantly larger than those of the PNH (*p* < 0.01) and fibrin gel groups (*p* < 0.001) and the suture group (*p* < 0.001) (Figure 6g). Infiltration of large inflammatory cells was observed in the suture, fibrin gel, and PNH groups; in contrast, the number of inflammatory cells in the hybrid patch group was significantly lower than that in the other groups (*p* < 0.001, Figure 6h,i). The influence of the four treatments on neovascularization of the perforated area was evaluated using immunofluorescence staining against CD31, which is specifically expressed in endothelial cells and is a marker of angiogenesis. As shown in Figure 6f,k, the number of blood vessels present in the hybrid patch group was significantly higher than that in the fibrin gel (*p* < 0.001) and suture group (*p* < 0.01). The results indicated that the hybrid patch could be used as a powerful sealant for inhibiting the leakage of gastric fluid, avoiding undesired adhesion with adjacent tissues after surgery, and promoting healing of the perforated stomach in vivo. Therefore, the structural color medical patch with dual surface properties of wet bioadhesion and slipperiness is a perfect biomaterial, not only for the repair of gastric perforation, but also for various tissue defects.

## 3 Conclusion

In summary, we presented a novel multilayer wet bioadhesive, with anisotropic surface adhesion, for internal tissue repair and for avoiding undesired adhesion to adjacent tissues after surgery. PAAc–PEG–NHS/gelatin hydrogels and liquid paraffin were used as the wet adhesive and slippery layers, respectively, which were infused into the two sides of a PLGA inverse opal scaffold, to form an integral multilayer patch. The multilayer patch demonstrated strong adhesion to wet porcine skin, stomach, and intestine and this was evaluated through the measurement of the shear stress, interfacial toughness, and tensile stress between the patch and tissues. It was proven that when applied in a rat model with full‐thickness gastric perforation, the designed multilayer wet bioadhesive with anisotropic adhesion could adhere tightly to the defective tissue and improve tissue repair without any adhesion to the adjacent tissues. These features endow the new wet bioadhesive with great potential for the tissue repair and regenerative medicine.

## 4 Experimental Section

### Materials

All chemical materials not mentioned were bought from Sigma‐Aldrich (USA). aa–PEG–NHS was provided by Ponsure biological Corporation (Shanghai, China). The commercially available sealants Dermabond and Bioseal were provided by Nanjing Drum Tower Hospital. The silica nanoparticles were self‐prepared. PLGA with the molecular weight of 8 w Da was purchased from Jinan Daigang Biomaterials Corporation. Liquid paraffin was provided from Nanjing Jinling Hospital. The tissues used were bought from the local farmer's market. NIH‐3T3 cells were provided by the Chinese Academy of Sciences. The cell culture‐related materials were bought from Gibco (USA). The cell Live–Dead assay kit and CCK‐8 were purchased from KeyGen Biotech Corporation (Jiangsu, China). The primary antibody of Ki67, IL‐6, TNF‐*α*, and CD31, and secondary antibody of Alexa Fluor 594, HRP‐conjugated goat anti‐rabbit IgG H&L were bought from Abcam (USA). The 6 weeks male SD rats were bought from the Model Animal Research Center of Nanjing University.

### Fabrication and Characterization of the Wet Adhesive PNH

The PNH was prepared based on a previously reported method with a modification.^[^ ^17^ ^]^ In brief, 30% aa, 10% gelatin, 1% aa–PEG–NHS, 0.1% BIS, and 1% HMPP (w/w) were dissolved in distilled water under magnetic stirring at 37 °C for 2 h. The pregel solution was then exposed to the UV light for 30 min to form the wet adhesive PNH, which was further totally dried in the hood for 24 h to obtain the dry PNH.^[^ ^1^ ^]^ HNMR (Bruker 600 MHz) and mass spectrum (Bruker autoflex maX MALDI‐TOF) was carried out to measure the molecular weight of aa–PEG–NHS. FTIR spectrometer (Thermo Fisher, IN10) was used to obtain spectra to determine the chemical functional groups of PNH. GPC (Agilent PL‐GPC50) and TG (NETZSCH STA 449 F5) were performed to determine the molecular weight and thermal stability of the PNH.

### Measurement of Adhesive Properties

The shear strength, interfacial toughness, and tensile strength of PNH/dry PNH on the porcine skin were measured through the standard lap‐shear test (ASTM F2255), 180‐degree peel test (ASTM F2256), and tensile test (ASTM F2258), respectively, by using tensile machine (INSTRON, Germany). The commercially available sealants Dermabond and Bioseal were used as control. Transparent poly(methyl methacrylate) film was used as the stiff backing of the tissue during the mechanical test. Notably, the PNH was directly adhered to the tissues followed by 20 s of pressing, but for the dry PNH, a little amount of PBS was added to the tissues to trigger the adhesion before pressing. For the measurement of shear strength, the rectangular porcine skin with the width and length of 10 and 40 mm was prepared. The sealants were added between two tissues with an adhesion area of 15 mm by 10 mm. The shear strength was calculated by the following formula: shear strength = *F*_max_/adhesion area, where *F*_max_ was the maximum force. For the measurement of interfacial toughness, the rectangular porcine skin with the width and length of 10 and 40 mm was prepared. The sealants were added between two tissues with an adhesion area of 10 mm by 10 mm. The interfacial toughness was calculated by the following formula: interfacial toughness = 2*F*_max_/*w*, where *F*_max_ was the maximum force and *w* was the width of the adhesion area. For the measurement of tensile strength, the square porcine skin with the width and length of 20 mm was prepared. The sealants were added between two tissues with an adhesion area of 20 mm by 20 mm. The tensile strength was calculated by the following formula: tensile strength = *F*_max_/adhesion area, where *F*_max_ was the maximum force. Three samples were measured for each condition to assure the reliability of the data.

### Measurement of Biodegradation, Swelling Ratio, Viscoelastic Properties, and Microscopic Structure

For the measurement of biodegradation, buffer with different pH of 7, 5, and 3 was prepared by blending disodium hydrogen phosphate and citric acid. To prepare in vitro enzymatic biodegradation medium, 10 mg collagenase was added in 100 mL buffer with pH of 7. The cylindrical PNHs with the diameter height of 3 mm and diameter of 5 mm were fabricated and accurately weighed after freeze‐drying. Then, each sample was immersed in 15 mL of the buffers with different pH and enzymatic medium and incubated at 37 °C with shaking at 60 rpm. At each time interval, the PNH was removed from the incubation medium, washed with deionized water, and lyophilized. Weight loss was determined as the percentage ratio of the mass of the lyophilized sample at each time interval, normalized by the dry mass of the original lyophilized sample.

For the measurement of the swelling ratio, the cylindrical PNHs with the diameter height of 5 mm were fabricated. The PNHs were immersed into PBS, SGF, and SIF at 37 °C for 1, 2, 4, 8, 16, 24, 48, and 72 h, respectively. The wet weight of the samples at each time point was measured after wiping off the redundant water by Kimwipe paper. Then, the samples were frozen quickly in the liquid nitrogen and dried in a lyophilizer, followed by the measurement of the dry weight. The swelling ratios were measured through the division of the dry weight by the wet weight of the samples. Three samples were measured for each condition to assure the reliability of the data.

For the measurement of viscoelastic properties, a hybrid rheometer (Discovery HR‐1, USA) was used. The PNHs were immersed into PBS, SGF, and SIF at 37 °C for 24 h, and all samples were cut into a cylinder with diameter and height of 20 and 2 mm to match the clamp of the rheometer. The swelling of the PNH in the fluids would lead to the surface of the samples being slippery. To avoid the slipping of the samples during measurement, the Kimwipe paper was used to wrap up the sample. The oscillatory frequency sweep for all samples were carried out at a fixed constant strain of 1% with a frequency ranging from 0.1 to 400 Hz, while the oscillatory strain sweep for all samples were performed at a constant frequency of 1 Hz.

To observe the microscopic structure of the hydrogels, PNHs were immersed into PBS, SGF, and SIF at 37 °C for 24 h. All samples were frozen quickly in the liquid nitrogen and dried in a lyophilizer, followed by the coating of a 20‐nm‐thick layer of gold. The morphology of these freeze‐dried samples was imaged using a SEM (ZEISS, Germany). The pore size of the samples was measured using the software of ImageJ.

### Fabrication of the Hybrid Patch

The PLGA solution with the concentration of 20% w/v was obtained by dissolving PLGA into chloroform solvent under magnetic stirring at 50 °C overnight. The silica colloidal crystal template was prepared by the self‐assembly of silicon dioxide (SiO_2_) nanoparticles on a glass slide with the evaporation of the solvent. The PLGA solution was perforated into the space between two colloidal crystal templates with a distance of 100 µm. With the evaporation of chloroform for 5 days in the hood, PLGA could be solidified totally. Then, hydrofluoric acid with the concentration of 4% w/v was prepared for the etching of SiO_2_ nanoparticle for 2 h to fabricate the PLGA scaffold with double layers of inverse opal structure. Then, liquid paraffin was first filled to one side of the inverse opal scaffold under the vacuum environment to form a lubricated surface. The PNH pregel solution was then infused into the other surface to generate the wet adhesive surface under UV light after plasma treatment.

### Characterization of the Hybrid Patch

The microscopic morphology of the colloidal crystal template, PLGA solution infused colloidal crystal template, PLGA scaffold with double layers of inverse opal structure, and the liquid paraffin infused inverse opal scaffolds were determined via SEM. The reflection spectra of the template, PLGA IO, and liquid paraffin infused PLGA IO, and the dynamic variation of the reflection spectra during the stretching of PLGA inverse opal scaffold and the hybrid patch was measured using a fiber optic spectrometer (Ocean Optics, USB2000‐FLG). To measure the mechanical properties, rectangular PLGA scaffold, PLGA inverse opal scaffold, and hybrid patch with the length, width, and thickness of 50, 30 mm and 200 µm were prepared. The maximum tensile stress and strain were recorded by the mechanical testing machine. To measure the tensile strength on the porcine skin, the rectangular PNH, hybrid patch without inverse opal structure, and hybrid patch with the length, width, and thickness of 20, 10 mm and 200 µm were fabricated. The tensile strain–stress curves, maximum tensile strength, and strain were recorded by using the mechanical testing machine.

### In Vitro Biocompatibility Evaluation

The extracts of the hybrid patch were prepared by immersing a certain amount of patch into the culture media for 24 h at 37 °C. The 3T3 cells were cultured for 24, 48, and 72 h in the extracts with different concentrations (10, 20, and 30 mg mL^−1^), respectively, and the cells cultured under normal culture media were used as control. At each time point, a Live/Dead assay was performed by staining the cells with the mixture of ethidium homodimer and calcein‐AM solution, followed by the observation by a fluorescent microscope (Leica, Germany). The cell viability was measured by using the CCK‐8 (DOJINDO, Japan). In brief, at each time point, the cells were incubated in the mixture of CCK‐8 solution and culture media for 1.5 h, followed by measuring the optical density at 450 nm via a plate reader. Three samples were measured for each condition to assure the reliability of the data.

### Characterization of the Wet Adhesive and Slippery Properties of the PNH

The 180‐degree peel test was carried out to determine the adhesive interfacial toughness of the hybrid patch on the wet porcine skin, stomach, and intestine immediately, 6, 12, 24, and 72 h after adhering using the mechanical testing machine. The rectangular hybrid patches with the length, width, and thickness of 20, 10 mm and 200 µm was fabricated and the adhesive surface at the two ends of the patch was adhered to the tissues with the length and width of 40 and 20 mm. The other end of these tissues was clamped by the machine. The interfacial toughness was calculated as 2*F*_max_/*w*, where *F*_max_ was the maximum force and *w* was the width of the patch. Three samples were measured for each condition to assure the reliability of the data.

To evaluate the slippery properties of the PNH, the water contact angles of the PLGA scaffold, PLGA IO, and liquid paraffin infused PLGA IO were measured based on a contact angle measuring system (JC2000D2) by using 2 µL water drop. The water sliding angles of the water drop with the volume of 6 µL on the PLGA IO and liquid paraffin‐infused PLGA IO was then measured.

### Animal Models

The animals were maintained in a temperature‐controlled environment (20 ± 1 °C) with free access to food and water. Animal care, breeding, and euthanasia were performed with the approval of the Animal Ethics Committee of Nanjing University (Approval number: 2021AE02007). The 15 male SD rats of 6‐week‐old were divided into five groups evenly. One group was used to determine the in vivo biocompatibility of the materials, in which the PNH and hybrid patch were performed subcutaneous implant to one rat, respectively, for 2 weeks, followed by the harvest of main tissues, including heart, liver, spleen, lung, and kidney for further evaluation. Other four groups were used to perform gastric perforation and sealing after fasting the rats for 36 h. A perforation with the diameter of 3 mm at the fundus of the stomach was generated by using a surgical trephine; 8‐0 sutures, 100 µL fibrin gel, cylindrical PNH, and hybrid patch with the diameter of 6.5 mm and thickness of 200 µm were prepared to seal the perforation, respectively, in four groups. All surgeries were carried out under sterile environments. 24 h after surgery, the rats resumed a normal diet and were taken care and monitored according to standards. Immediately and 7 days after the operation, the sealed and repaired conditions of the stomach were observed and photographed by a digital camera, followed by the sacrifice of the rats to obtain the gastric tissues around the sealed area, and main tissues for further evaluation. Besides, 100 µL of blood was collected from rat eyes and added to an EDTA‐K2 anticoagulation tube for blood routine examination. An additional 1.5 mL of blood was added to a procoagulant collection tube and the serum was obtained after centrifugation at 3500 × *g* for 5–10 min for biochemical analysis. An additional observation on day 3 was performed for the hybrid patch to determine the variation of structural color.

### Histology, Immunohistochemistry, and Immunofluorescence Staining

All tissues obtained were fixed into 4% formaldehyde solution, then dehydrated into a series of gradient ethanol before vitrification by dimethylbenzene. The treated tissues were embedded in paraffin and sliced into slides with a thickness of 5 µm by a microtome. For HE and MASSON staining, the slices were deparaffinized, rehydrated, and then immersed into the specific staining solutions according to the manufacturer's manual, respectively. For immunohistochemistry analysis, the slices were deparaffinized, rehydrated, and then incubated with primary antibodies (Ki67, IL‐6, and TNF‐*α*) and secondary antibodies, followed by the staining with DAB and hematoxylin. For immunofluorescence staining, the slides were deparaffinized, rehydrated, and then incubated with primary antibodies (CD31) and secondary antibodies, then, the samples were mounted by 4,6‐diamidino‐2‐phenylindole solution. The slides were observed under fluorescent microscopy (Leica, Germany).

### Statistical Analysis

All the results were presented as the means ± standard deviation. Statistical analysis was carried out using Student's *t*‐test or one‐way ANOVA followed by Tukey's post‐hoc test to determine the degree of significance by the software of Origin 2022. Statistical significance was defined as *p < 0.05, **p < 0.01, ***p < 0.001.

## Conflict of Interest

The authors declare no conflict of interest.

## Author Contributions

Y.J.Z. conceived the idea and designed the experiment; B.K., R.L., and Y.C. conducted experiments; B.K. and R.L. performed data analysis; B.K. and Y.J.Z. wrote the manuscript. Y.X.S., D.G.Z., H.C.G., and W.X. contributed to scientific discussion of the article.

## Supporting information

Supporting Information

Supplemental Movie 1

## Data Availability

The data that support the findings of this study are available from the corresponding author upon reasonable request.
