# Supplementary material for: Structural Color Medical Patch with Surface Dual‐Properties of Wet Bioadhesion and Slipperiness
Source: Adv Sci (Weinh). 2022 Sep 11;9(31):2203096. doi: 10.1002/advs.202203096 (PMC9631070; doi:10.1002/advs.202203096)
Supplement: Supplementary file 1 — Supporting Information [file ADVS-9-2203096-s002.pdf]

ADVANCED SCIENCE
Open Access

Supporting Information

for *Adv. Sci.*, DOI 10.1002/advs.202203096

Structural Color Medical Patch with Surface Dual-Properties of Wet Bioadhesion and Slipperiness

Bin Kong, Rui Liu, Yi Cheng, Yixuan Shang, Dagan Zhang, Hongcheng Gu, Yuanjin Zhao* and Wei Xu**

Supplementary

Structural color medical patch with surface dual-properties of wet bioadhesion and slipperiness

Bin Kong [a], Rui Liu [a], Yi Cheng [a], Yixuan Shang [a], Dagan Zhang [a], Hongcheng Gu [a,*], Wei Xu [a,b,*], Yuanjin Zhao [a,c,*]

a. *Department of Rheumatology and Immunology, Nanjing Drum Tower Hospital, School of Biological Science and Medical Engineering, Southeast University, Nanjing 210096, China*

b. *Department of Orthopedics, Tongren Hospital, Shanghai Jiao Tong University School of Medicine, Shanghai 200336, China*

c. *Oujiang Laboratory (Zhejiang Lab for Regenerative Medicine, Vision and Brain Health), Wenzhou Institute, University of Chinese Academy of Sciences, Wenzhou, Zhejiang 325001, China*

Email: weixu@shsmu.edu.cn (W.X.); hcgu@seu.edu.cn (H.C.G.); yjzhao@seu.edu.cn (Y.J.Z.)

Figures

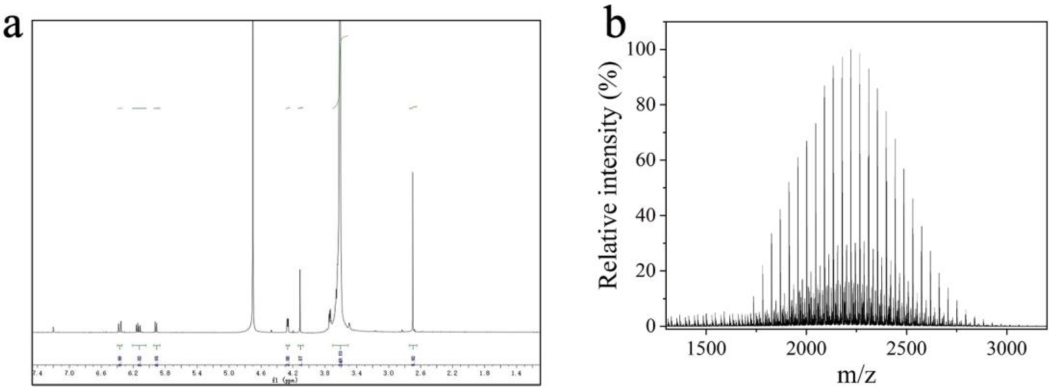

Figure S1. The ^{1}HNMR (a) and mass spectrum (b) of aa-PEG-NHS.

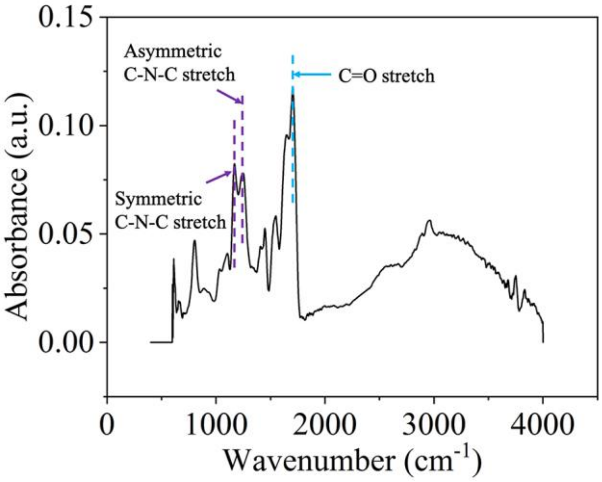

Figure S2. The transmittance FTIR spectrum.

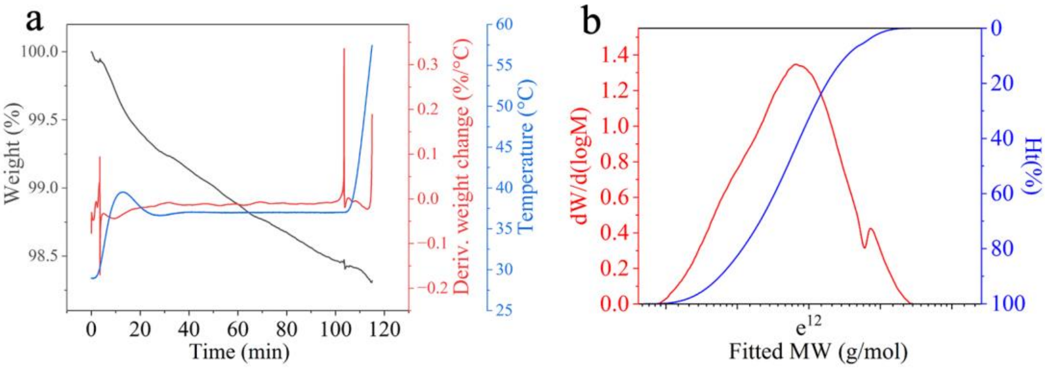

Figure S3. TG (a) and GPC (b) analysis of the PNH.

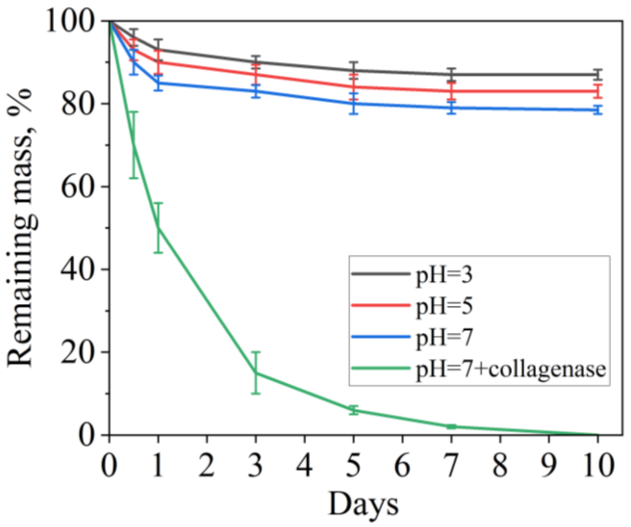

Figure S4. *In vitro* biodegradation of the PNH in the buffer of different pH with or without collagenase.

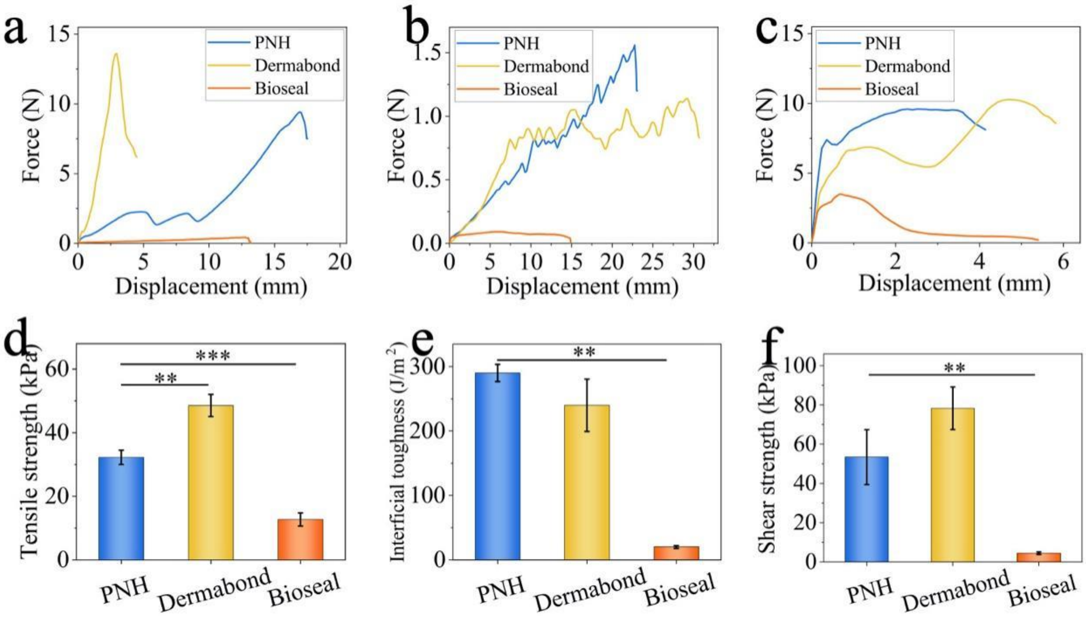

Figure S5. Measurement of shear strength (a), (d), interfacial toughness (b), (e) and tensile strength (c), (f) of wet PNH, Dermabond and Bioseal on the porcine skin.

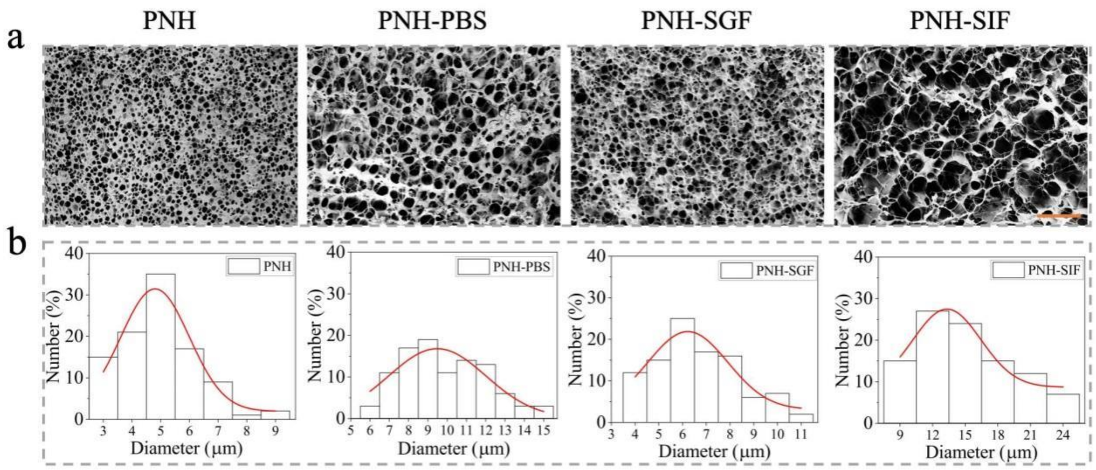

Figure S6. (a) The SEM images showing the microscopic structures of PNH, and PNH after immersing into PBS, SGF and SIF, respectively. (b) The corresponding distribution of the pore size. The scale bar is 50 μm.

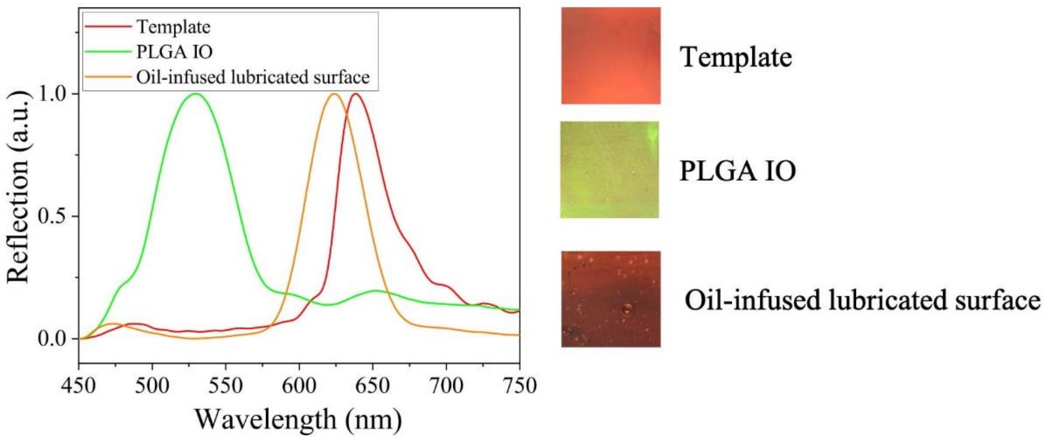

Figure S7. The reflection spectra of the colloidal crystal template, PLGA inverse opal scaffold and liquid paraffin infused hybrid patch.

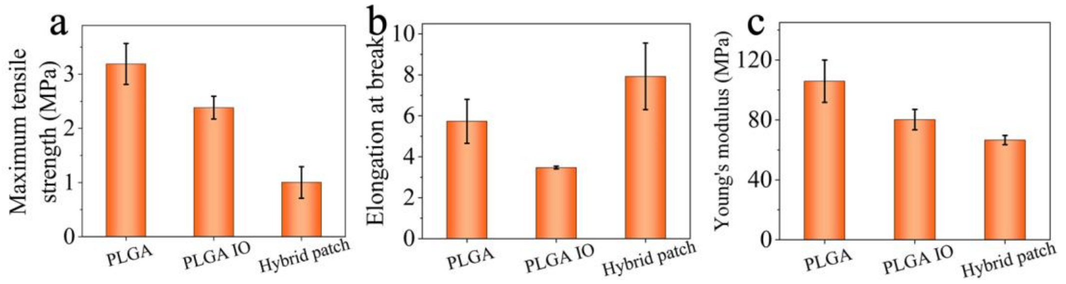

Figure S8. The histogram of the tensile strength and strain of the PLGA scaffold, PLGA inverse opal scaffold and hybrid patch.

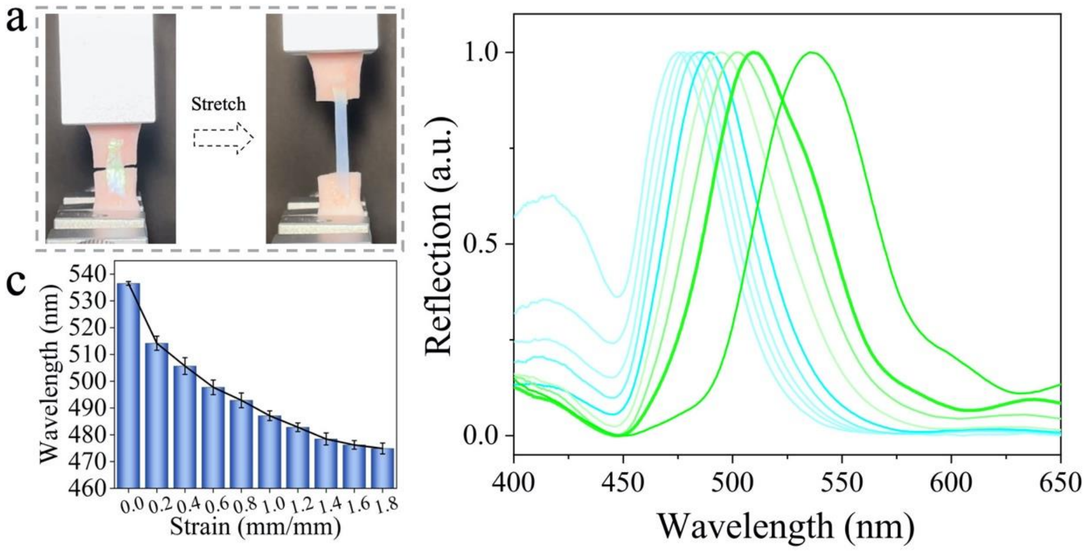

Figure S9. (a) Mechanical responsive color variation of the hybrid patch without oil infusion adhered on porcine skin. (b) Corresponding reflection spectra change. (c) The peak position of the reflection spectra as the function of strain.

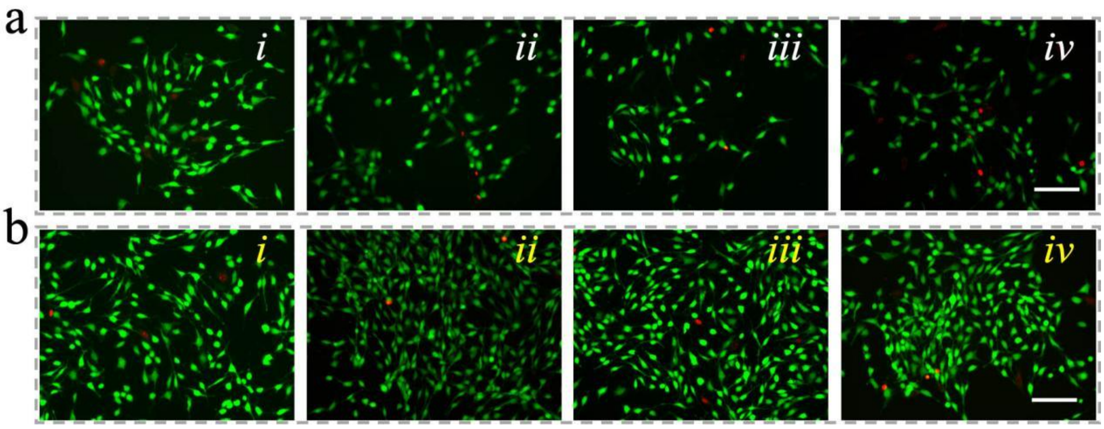

Figure S10. The Live/Dead staining images of 3T3 cells after culturing in the extracted liquid of the hybrid patch with the concentrations of i) 0, ii) 10, iii) 20 and iv) 30 mg/mL for 24 h and 48 h. The scale bar is 100 μm.

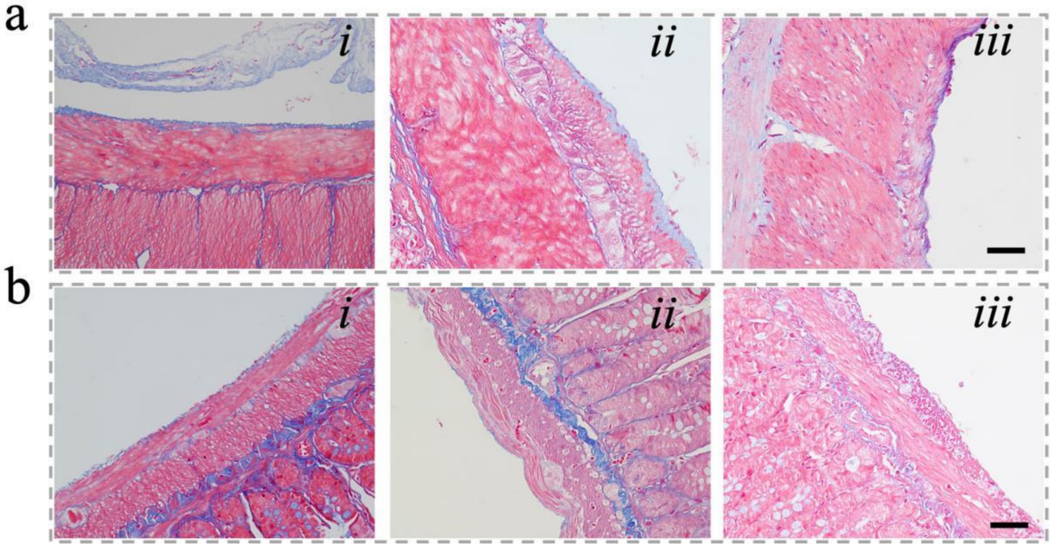

Figure S11. (a) The HE staining images of i) normal stomach; ii) stomach adhered with the hybrid patch; iii) stomach adhered with the hybrid patch after immersing into SGF for 4 h. (b) The HE staining images of i) normal intestine; ii) intestine adhered with the hybrid patch; iii) intestine adhered with the hybrid patch after immersing into SIF for 4 h. The scale bar is 100 μm.

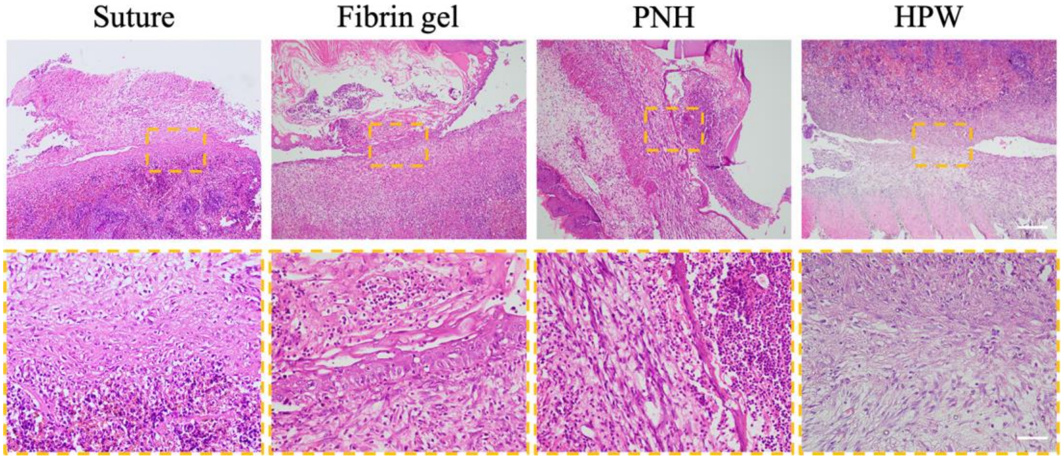

Figure S12. Representative HE staining images showing the postoperative adhesion of the stomach with adjacent tissues on the group of sutures, fibrin gel and PNH. The scale bar is 200 μm and 50 μm in the enlarged images.

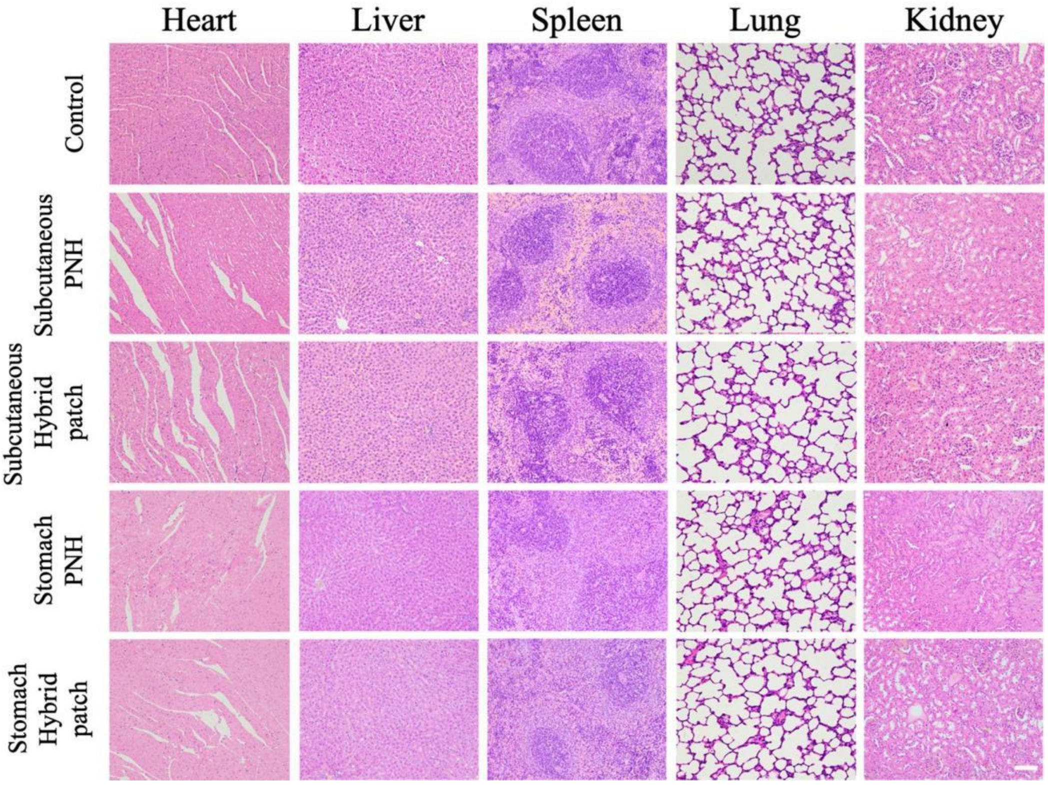

Figure S13. Representative HE staining images of heart, liver, spleen, lung and kidney from normal rats (control), rats subcutaneously implanted with PNH and hybrid patch for 2 weeks respectively, and rats implanted with PNH and hybrid patch on the stomach for 7 days respectively. The scale bar is 100 μm.

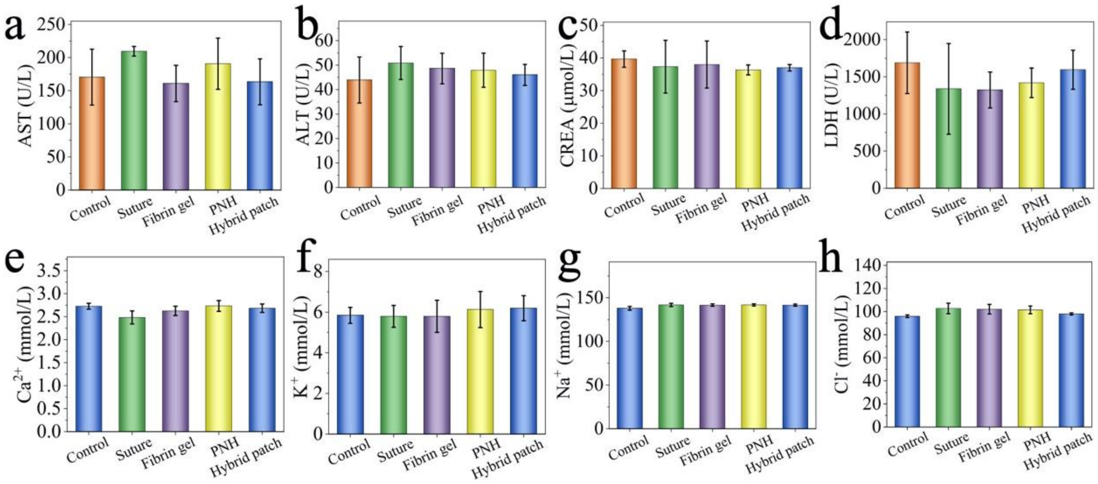

Figure S14. The biochemical analysis of the blood from the normal rats (control), rats sealed with suture, fibrin gel, PNH and hybrid patch.

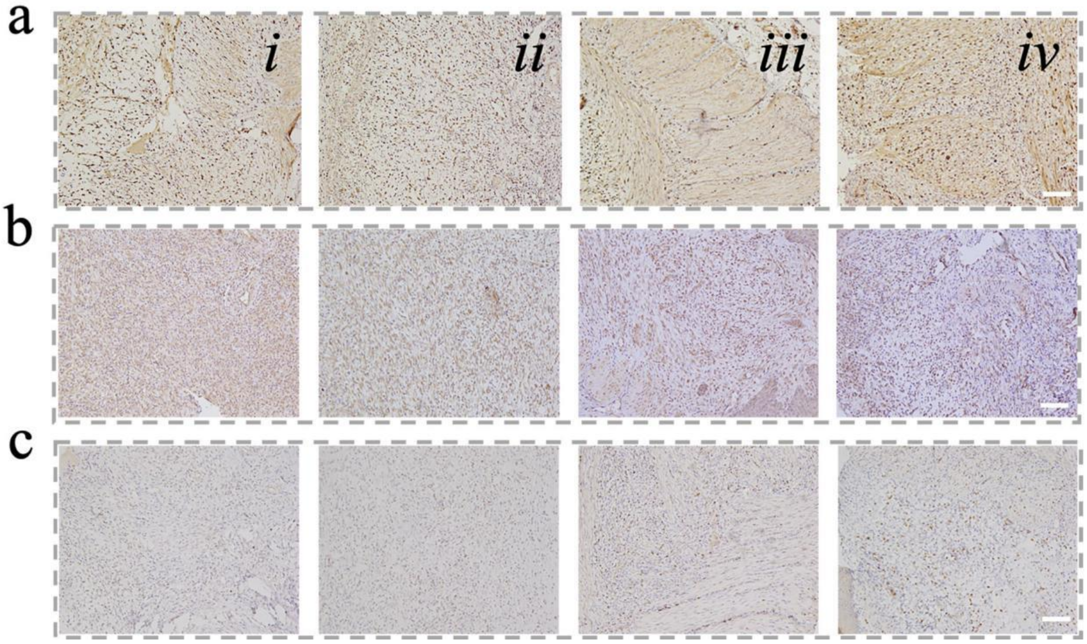

Figure S15. Immunostaining of (a) Ki67, (b) TNF-α and (c) IL-6 in the wound area 7 days after treated with i) suture, ii) fibrin gel, iii) PNH and iv) hybrid patch. The scale bar is 100 μm.
